# Between chromatin and SNPs: genetic variability and the susceptibility to acute kidney injury

**DOI:** 10.1186/s13054-017-1716-z

**Published:** 2017-06-06

**Authors:** Hernando Gómez

**Affiliations:** Center for Critical Care Nephrology, Department of Critical Care Medicine, University of Pittsburgh, School of Medicine, 3347 Forbes Ave., Suite 220, Rm 207, Pittsburgh, PA 15213 USA

**Keywords:** Liver, Transplantation, Acute kidney injury, AKI, Albumin, Hypoalbuminemia, Mortality

Acute kidney injury (AKI) is a common and deadly condition that is particularly frequent in the injured, the surgical, and the critically ill patient [1]. Regardless of its severity, AKI increases the risk of death [2] and in survivors represents a significant burden because it is directly associated with the development of chronic kidney disease [3]. Despite the highly relevant consequences AKI carries, the mechanisms by which different insults lead to the clinical syndrome we know as acute kidney injury are incompletely understood.

Injury to the renal epithelium and the response of the tubular epithelial cell (TEC) to such injury are key components to understand the mechanisms leading to AKI. Zager [4] provided evidence to suggest that, after mounting a defense response, the renal epithelium is capable of “remembering” this insult and using this information to modulate future responses (i.e., engage a more robust inflammatory response). This “memory” mechanism was shown to be dependent on epigenetic regulation through chromatin-remodeling enzymes (i.e., histone deacetylaces or HDACs) that ultimately re-program the TECs to respond to injury [4]. These data suggest that genetic modulation is at the forefront of the cellular response and that genetic alterations are, therefore, likely to be a determinant factor of susceptibility.

Xu and collaborators [5] provided evidence of the fundamental role of genetic control in the development of AKI. The authors demonstrated that despite resulting in similar severity of AKI, ischemia/reperfusion and hypovolemia triggered completely different genetic programs. These data demonstrate that AKI is not a unique disease, but rather a heterogeneous syndrome, and that variations in gene expression may be key to understanding the governing mechanisms leading to AKI.

The most common genetic variation in humans is due to single nucleotide polymorphisms (SNPs), which occur when a single nucleotide is changed at a specific position within the genome. SNPs have been shown to be associated with susceptibility to a wide range of conditions. The first study to explore the association of potential genetic polymorphisms with sepsis-induced AKI using large-scale gene-centric genotyping was done by Frank and collaborators [6]. They identified five SNPs associated with AKI, three of which were in genes related to apoptotic pathways: SERPINA4, SERPINA5, and BCL2. Expression of these genes was associated with decreased risk of AKI with odds ratios of 0.55, 0.57, and 0.61 (*p* < 0.05), respectively. Three systematic reviews of the literature have addressed this question [7–9], all reaching similar conclusions: despite finding several associations, the included studies were of low to moderate quality and were significantly heterogeneous, precluding any definitive conclusion.

In this issue of *Critical Care*, Vilander et al. [10] present an important study to the field. The authors conducted the study in 837 patients (without chronic kidney disease) with severe sepsis enrolled in the FINNAKI study. They found that only SNPs in the SERPINA4 and SERPINA5 genes were associated with AKI KDIGO stages 2 and 3 in septic shock patients, but not patients with severe sepsis. This is key because it lends support to the notion that, even in the context of a similar originating insult (i.e., sepsis), susceptibility to AKI may be related to different mechanisms in different subgroups of patients. The study by Vilander et al. strengthens the findings by Frank et al. in a similar patient population, and lays the ground for future work to unveil the mechanistic underpinnings of these associations.

In conclusion, genetic variation in the form of SNPs or chromatin remodeling seems to have a fundamental role in conferring susceptibility to AKI. The thought-provoking work by Frank et al. and Vilander et al. has paved the way for mechanistic studies to further understand these interactions. However, it will be critical to avoid the mistake of considering AKI or sepsis as “single-gene” diseases and to embrace them as what they have always been, syndromes.

## Abbreviations

AKI: Acute kidney injury
SNP: Single nucleotide polymorphism
TEC: tubular epithelial cell
